# Synthesis of Block Copolymers by Mechanistic Transformation from Reversible Complexation Mediated Living Radical Polymerization to the Photoinduced Radical Oxidation/Addition/Deactivation Process

**DOI:** 10.1021/acsmacrolett.2c00004

**Published:** 2022-02-22

**Authors:** Cansu Aydogan, F. Simal Aykac, Gorkem Yilmaz, Ye Qiu Chew, Atsushi Goto, Yusuf Yagci

**Affiliations:** † Department of Chemistry, Faculty of Science and Letters, Istanbul Technical University, 34469 Maslak, Istanbul, Turkey; ‡ Division of Chemistry and Biological Chemistry, School of Physical and Mathematical Sciences, Nanyang Technological University, 21 Nanyang Link, 637371 Singapore; § Faculty of Science, Chemistry Department, King Abdulaziz University, 21589 Jeddah, Saudi Arabia

## Abstract

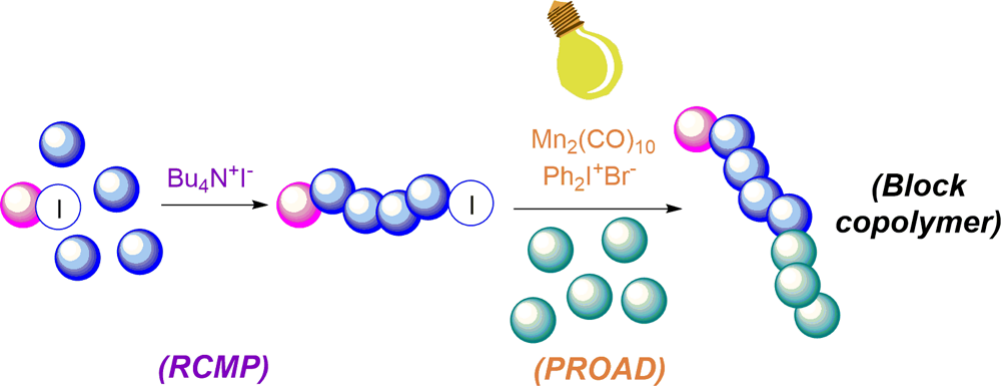

A versatile strategy for the fabrication of block copolymers by the combination of two discrete living polymerization techniques—reversible complexation mediated living radical polymerization (RCMP) and photoinduced radical oxidation addition deactivation (PROAD) processes—is reported. First, RCMP is conducted to yield poly(methyl methacrylate) with iodide end groups (PMMA-I). In the following step, PMMA-I is used as macroinitiator for living PROAD cationic polymerization of isobutyl vinyl ether. Successful formation of the block copolymers is confirmed by ^1^H NMR, FT-IR, GPC, and DSC investigations.

Recently, block copolymers have been the subject of detailed investigations as they are excellent candidates for various applications such as adhesives, drug delivery, nanomedicine, soft lithography, and thermoplastic elastomers due to their range of desirable properties emerging from each discrete monomer segments.^1−7^ The possibility of adjusting structural and compositional versatility of each segments to tune the physicochemical properties on demand has pushed the research frontiers toward the development of novel synthetic strategies for the preparation of block copolymers.^8−11^ Traditionally, anionic living polymerization (LP) was employed for the synthesis of block copolymers using the sequential monomer addition technique. However, the current research progress is closely related to contemporary radical polymerization methods as well as cationic polymerization as they require easy experimentation procedures.^12−19^

In recent years, mechanistic transformation techniques has been widely employed for expanding the scope of variations of block or graft copolymers by the combination of different polymerization modes. Mechanistic transformations were successfully applied to all types of addition polymerizations: radical, cationic, and anionic polymerizations.^28,29^ By use of this approach, block copolymers that cannot be obtained by a single polymerization mode can be prepared efficiently. Mechanistic transformations can be realized by two distinct strategies, namely direct and indirect transformation reactions. Direct transformation refers the transformation of a propagating active center to another active center with different polarity. In general, such transformation occurs through electron transfer process. The indirect transformation technique considers introduction of the stable but potentially reactive functional group for the second polymerization mode at the chain ends, either in the initiation or in the termination steps of the polymerization of the first monomer. After the isolation and purification of the polymer, finally the functional groups are converted to another species.^30^ Intriguing developments on controlled/living polymerizations by radical and cationic mechanisms with the capability to control over functional groups and molecular weight opened new routes for mechanistic transformations.^31−39^

Controlled/living radical polymerization (CLRP), also called reversible deactivation radical polymerization (RDRP), has widely been used as an efficient tool for the synthesis of well-defined polymers with low dispersity. The mechanism is grounded on the reversible activation of the dormant species (Polymer-X) to the propagating radical (Polymer^•^) using certain additives. The most common LRP techniques are atom transfer radical polymerization^40,41^ (ATRP), reversible addition–fragmentation transfer polymerization,^42,43^ and nitroxide-mediated polymerization.^44^ Goto and co-workers presented a novel method coined “reversible complexation mediated polymerization’’ (RCMP) for the synthesis of polymers with well-defined structures.^45−48^ It considers the use of alkyl iodides as initiator and organic salts such as tetrabutylammonium iodide as catalyst, which provides reversible generation of R^•^. The catalyst activates Polymer-I to form Polymer^•^ and A^+^I_2_^•–^ (Scheme 1). After incorporation of a few monomers, Polymer^•^ is deactivated by the radicalic salt catalyst to generate Polymer-I and A^+^I^–^ again. Polymer-I is also activated to Polymer^•^ via degenerative chain transfer.^49^ The contributions of the catalytic process and degenerative chain transfer depend on the systems.^50^ By repeated activation–deactivation cycles, the polymer grows gradually, resulting in the formation of polymers with low polydispersity (Scheme 1).

**Scheme 1:**
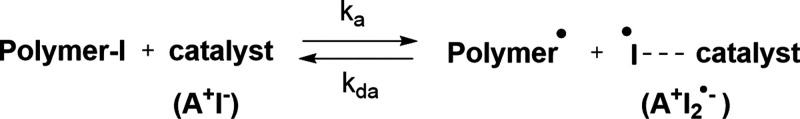
General Mechanism of RCMP

Although cationic polymerization has been utilized for industrial applications for decades, bringing controlled/living features to cationic polymerization remained a challenge until Higashimura et al.^51^ as well as Kennedy and Faust^52^ independently reported the living cationic polymerization of vinyl ethers and isobutylene, respectively.

Kamigaito et al. reported the living cationic polymerization of vinyl ethers using acetic acid derivatives and Lewis acids as initiators.^53^ The technique was improved by the use of zinc halides as additives to regulate the nucleophilicity of the chain ends, which overcome the necessity of using acids with nonnucleophilic counterparts such as PF_6_^–^, SbF_6_^–^, and BF_4_^–^. Adaptation of this approach to a photochemical strategy has later been demonstrated by Yagci and co-workers for the preparation of poly(vinyl ether)s with controlled molecular weight characteristics.^54^

Photoinduced radical oxidation addition deactivation (PROAD) processes have recently become an alternative way for the living cationic polymerization of vinyl ethers. The mechanism follows visible light induced halide abstraction followed by oxidation, addition, and deactivation processes using alkyl halide, Mn_2_(CO)_10_, and diphenyliodonium bromide (Ph_2_I^+^Br^–^) as the onium salt^55^ (Scheme 2).

**Scheme 2:**
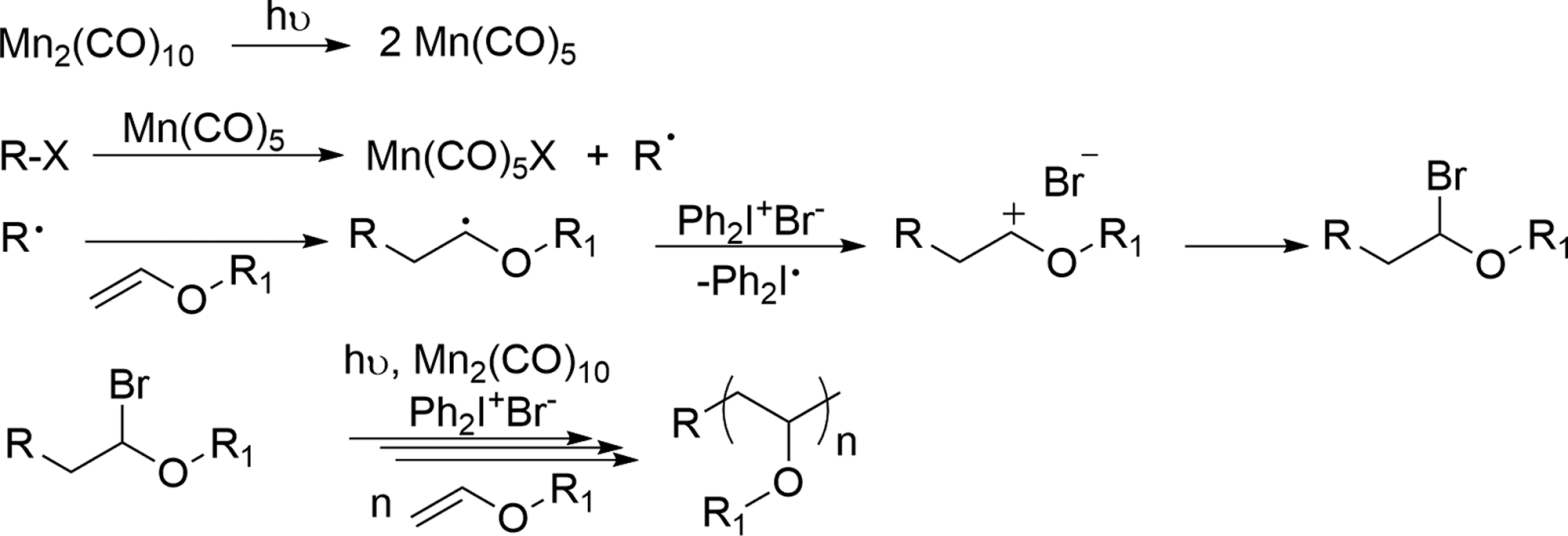
Mechanism of PROAD Polymerization of Vinyl Ethers

Facilitated by the chain-end fidelity, PROAD methodology was exploited for the realization of various mechanistic transformation reactions. For instance, block copolymers from cationically polymerizable vinyl ethers with vinyl monomers polymerizable by radical means were prepared by combining iniferter with PROAD processes proceeding in a controlled manner.^56^ In another study, two discrete living polymerization were united for the preparation of amphiphilic block copolymers. The bromo end group of poly(methyl methacrylate) prepared by ATRP was converted to the triphenylmethyl (trityl) functionality by visible light induced simultaneous halide abstraction and coupling reactions. The obtained polymers bearing trityl end groups acted as macroiniferters which enabled the polymerization of vinyl monomers to yield desired block copolymers in a controlled fashion. Poly(*tert-*butyl acrylate)-based block copolymers are essentially converted to poly(acrylic acid), resulting in the formation of amphiphilic copolymers with a facile hydrolization protocol.^57^ The current work was designed to combine RCMP with PROAD processes both proceeding in a controlled manner to produce block copolymers with well-defined structures by a mechanistic transformation pathway. The presented approach is particularly useful as the type of the monomers used are structurally different and cannot be combined by either mechanism.

We first performed RCMP of methyl methacrylate (MMA) (100 equiv) using ethyl 2-iodo-2-phenylacetate (EPh-I) (1 equiv) and tetrabutylammonium iodide (BNI) (1.5 equiv) at 60 °C for 2 h. To keep high iodide chain-end fidelity of the obtained polymer, we intentionally stopped the polymerization at a relatively short time (at a moderate monomer conversion of 31%). After purification via reprecipitation in hexane, we obtained a poly(methyl methacrylate)–iodide (PMMA-I) (*M*_n_ = 3900 and *M*_w_/*M*_n_ = 1.12 after purification). A chain extension test of PMMA-I in an RCMP of MMA demonstrated a relatively high iodide chain-end fidelity (≥86%) of PMMA-I (Supporting Information).

Poly(isobutyl vinyl ether) segments were photochemically attached to iodide chain-end polymers (PMMA-I) in the presence of Mn_2_(CO)_10_ in conjunction with an oxidant. In the process, PMMA-I served as macroinitiator and oxidized by Ph_2_I ^+^Br^–^ to give the corresponding carbocation capable of inducing cationic living polymerization of isobutyl vinyl ether (IBVE) under visible light irradiation (Scheme 3).

**Scheme 3:**
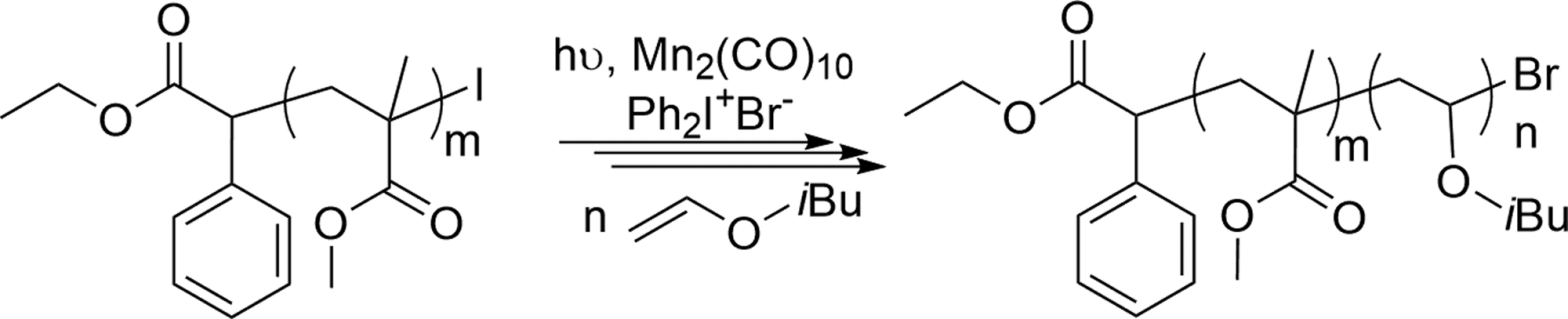
Synthesis of Block Copolymers by the Combination of RCMP and PROAD Polymerizations

The block copolymerizations of IBVE with PMMA-I were performed under different experimental conditions, and the results are tabulated in Table 1.

**Table 1:** PROAD Polymerization of IBVE Using PMMA-I as Precursor^a^

run	[Mn_2_(CO)_10_]/[Ph_2_I^+^Br^–^]	*M*_n,NMR_^b^ (g/mol)	*M*_n,GPC_^c^ (g/mol)	*M*_w_/*M*_n_^c^
1	0.25/0.25	12400	13900	1.57
2	0.25/0.5	11300	13000	1.56
3	0.5/0.25	11500	11700	1.70
4	0.1/0.25	13100	15000	1.61

a *M*_n(PMMA-I)_: 3900 g/mol; [IBVE]/[PMMA-I]: 100/1. Propylene carbonate (PC) was used as solvent (*V*_PC_/*V*_IBVE_: 2/1), irradiation time = 90 min.

b Determined by ^1^H NMR: calculated by comparing the integral area of the sum of methine proton of PIBVE and methyl ester of PMMA with methylene proton of PIBVE.

c Determined by gel permeation chromatography according to polystyrene standards.

The structure of the block copolymers was investigated by ^1^H NMR and FT-IR spectral analyses. The signals between 3 and 3.5 ppm in the ^1^H NMR spectrum (Figure 1) confirmed the successful attachment of IBVE segment to the precursor polymer. Similarly, FT-IR spectroscopy revealed the characteristic bands of the PMMA and the PIBVE segments. The sharp band at 1724 cm^–1^ can be attributed to C=O stretching of PMMA while the etheric C–O–C stretching band observable at 1074 cm^–1^ corresponds the incorporated PIBVE segment (Figure S1).

**Figure 1:**
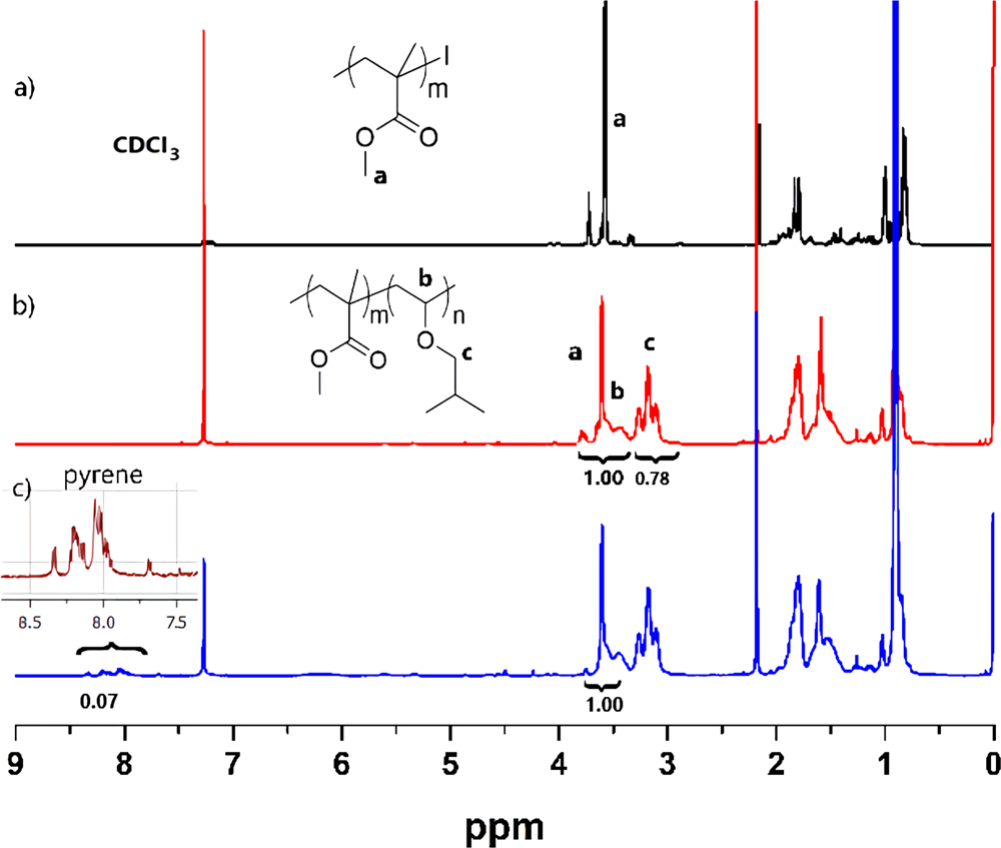
^1^H NMR spectra of (a) PMMA, (b) PMMA-*b*-PIBVE, and (c) pyrene functional PMMA-*b*-PIBVE.

Moreover, a clear shift to higher molecular weight region in GPC traces confirms successful block copolymerization process (Figure 2). Notably, no contamination of the precursor polymer was detected. The observed relatively broad dispersity can be attributed to slow addition of PMMA radical to vinyl ether as previously described.^38^

**Figure 2:**
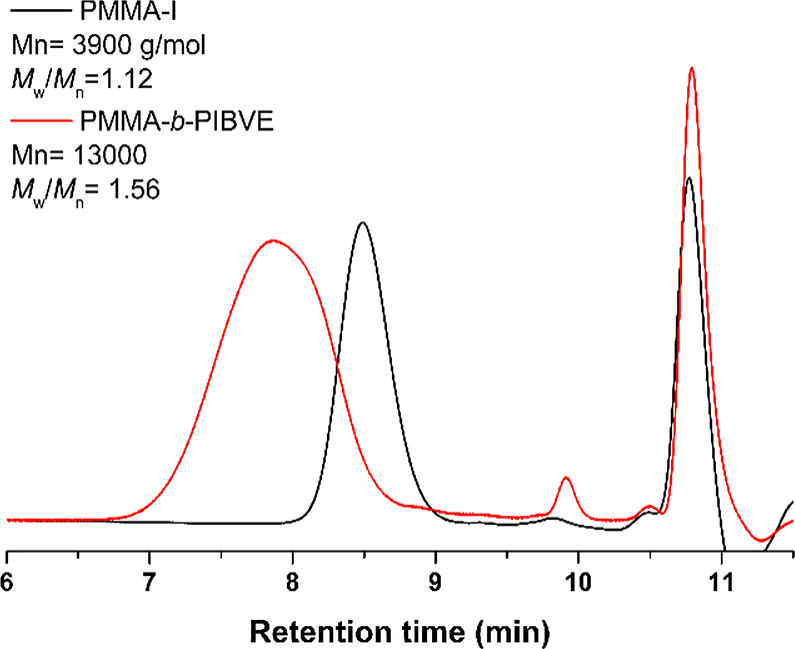
GPC traces of PMMA and PMMA-*b*-PIBVE.

Differential scanning calorimetry (DSC) investigation was further performed for the thermal characterization of the block copolymer. The glass transition peak of the pristine PMMA is detectable at 102 °C, while after block copolymerization, two glass transition temperatures were observed at −20 and 103 °C, corresponding to PIBVE and PMMA segments, respectively (Figure S2). This shows that the two segments are immiscible in this molecular weight composition.

The chain-end functionality of the block copolymers were not distinguishable probably due to the production of high molar mass polymers and/or the loss of the bromine functional group during the purification in methanol, which may lead to a substitution with methoxy groups.

To confirm the presence of bromide group at the chain end of polymers, the polymerization was terminated by adding pyrene-1-methanethiol to the reaction medium. This way, the chain end of the block copolymer was modified with pyrene groups by a simple substitution reaction. Then, thus-formed polymer was precipitated in methanol for purification. The incorporation of the pyrene moiety at the chain end was confirmed by NMR, UV, and fluorescence analyses. The aromatic peaks observed around 7.7–8.3 ppm in the ^1^H NMR spectrum of the block copolymer (Figure 1c, blue line) demonstrate the attachment of the aromatic rings. The strong absorption band above 300 nm in the UV spectrum (Figure S5a) and intense emission band above 400 nm in the fluorescence spectrum of the polymer (Figure S5b) clearly confirm the existence of the pyrene moieties at the chain ends of the polymer obtained. When the UV spectrum of the pyrene-attached block copolymer PMMA-*b*-PIBVE-pyrene was compared with that of bare pyrene at equal chromophore group concentrations, the absorption bands were found to be quite related (Figure S5a). Notably, polymer obtained without the functionalization process has no absorption (Figure S5a, blue line). To further prove the success of the chemical attachment of the pyrene group at the chain end and the absence of any unreacted pyrene residue, the molecular weights of PMMA-*b*-PIBVE-pyrene were calculated by NMR, GPC, and UV analyses and compared. Using the molar extinction coefficient of bare pyrene in THF, which was found as ε = 30000 L/(mol cm), the molecular weight of the block copolymer was calculated as 11700, which is in agreement with *M*_n,NMR_ and *M*_n,GPC_, 13100 and 15000 g/mol, respectively (run 4) The close values confirm successful functionalization and chain-end fidelity of the block copolymer.

In conclusion, a novel transformation system for the fabrication of well-defined block copolymers is proposed. This approach offers combination of two different living polymerization methods which allows to synthesize block copolymers with PMMA and PIBVE segments by sequential RCMP and PROAD processes, respectively. We believe that this strategy widened the scope of mechanistic transformation reactions and may serve a platform for the development of new complex architectures.

## Supporting Information Available

Materials, synthesis of the polymers, and additional spectral data (PDF)
