# Supplementary material for: Synthesis of Block Copolymers by Mechanistic Transformation from Reversible Complexation Mediated Living Radical Polymerization to the Photoinduced Radical Oxidation/Addition/Deactivation Process
Source: ACS Macro Lett. 2022 Feb 22;11(3):342–6. doi: 10.1021/acsmacrolett.2c00004 (PMC8928464; doi:10.1021/acsmacrolett.2c00004)
Supplement: Supplementary file 1 — mz2c00004_si_001.pdf [file mz2c00004_si_001.pdf]

Supporting Information

Synthesis of Block Copolymers by Mechanistic Transformation from Reversible Complexation Mediated Living Radical Polymerization to Photoinduced Radical Oxidation/Addition/ Deactivation Process

Cansu Aydogan [†], F.Simal Aykac[†],Gorkem Yilmaz[†], Ye Qiu Chew[‡], Atsushi Goto[‡,*], Yusuf Yagci[†,§ *]

[†]Department of Chemistry, Istanbul Technical University, Maslak, 34469 Istanbul, Turkey [‡]

[‡]Division of Chemistry and Biological Chemistry, School of Physical and Mathematical Sciences, Nanyang Technological University, 21 Nanyang Link, 637371, Singapore

[§]King Abdulaziz University, Faculty of Science, Chemistry Department, 21589 Jeddah, Saudi Arabia

*Corresponding author: agoto@ ntu.edu.sg; yusuf@itu.edu.tr

CONTENTS

Experimental Section

Materials

Synthesis of PMMA–I

Chain Extension PMMA–I

Synthesis of pyrene-1-methanethiol (Py-SH)

Synthesis of pyrene functional PMMA-*b*-PIBVE

Characterization

Figure S1. FTIR (ATR) spectra of (a) PMMA and (b) PMMA-b-PIBVE.

Figure S2. DSC traces of PMMA and PMMA-*b*-PIBVE.

Figure S3. GPC chromatograms for the polymer, PMMA–I (macroinitiator) before and after chain extention after 4 h.

Figure S4. ^{1}H NMR spectrum of Py-SH.

Figure S5. a) UV-vis spectra of PMMA-*b*-PIBVE (blue line); bare pyrene (red line) and PMMA-*b*-PIBVE-pyrene (black line). b) fluorescence emission spectrum of pyrene functional PMMA-*b*-PIBVE excited at 350 nm (5 x 10^{-5} mol L^{-1} in dichloromethane).

Experimental section

Materials

Methyl methacrylate (MMA) (>99.8%, Tokyo Chemical Industry (TCI), Japan), ethyl 2-iodo-2-phenylacetate (EPh–I) (>97.0%, TCI), and tetrabutylammonium iodide (BNI) (>98.0%, TCI) were used as received. Isobutylvinyl ether (IBVE, 99%, Aldrich) was vacuum-distilled from CaH_2. Diphenyliodonium bromide ($Ph_2I^+Br^-$, 97%, Aldrich), propylene carbonate (≥99.0%, Merck), tetrahydrofuran (THF, ≥99%, Aldrich) and methanol (99.9%, Merck) were used as received. Dimanganese decacarbonyl ($Mn_2(CO)_{10}$, 99%, Aldrich) was purified by sublimation and stored in a refrigerator in the dark.

Synthesis of PMMA–I

A mixture of MMA (25 g, 100 eq), EPh–I (1 eq), and BNI (1.5 eq) was heated in a 100 mL flask at 60 °C for 2 h under an argon atmosphere with magnetic stirring (monomer conversion = 31%). The reaction mixture was diluted with tetrahydrofuran (THF). The polymer was reprecipitated in hexane (non-solvent) and dried under vaccume for 5 h, yielding a PMMA–I (M_n = 3900 and M_w/M_n = 1.12).

Chain Extension PMMA–I

A mixture of MMA (1.01 g, 200 eq), PMMA–I (0.198 g, 1 eq), and BNI (0.0371 g, 2 eq) was heated in a Schlenk flask at 70 °C for 4 h under an argon atmosphere with magnetic stirring. The reaction mixtures at time zero and at 4 h were diluted with THF to know concentrations and analyzed using GPC. The GPC chromatograms (normalized by the concentrations) (Figure S2) showed that the PMMA–I (macroinitiator) peak decayed by 86% at 4 h. The result indicates the iodide-chain end fidelty of the PMMA–I (≥ 86%).

Synthesis of PMMA-*b*-PIBVE

An example of typical procedure is as follows; PMMA–I (1 eq) was dissolved in propylene carbonate (2 ml) and then, IBVE (100eq), $Ph_2I^+Br^-$ (0.25 eq) and $Mn_2(CO)_{10}$ (0.1 eq) were put into the Pyrex tube under dry nitrogen. The solution was then irradiated at room temperature for 90 mins by a Ker-Vis blue photoreactor which emits light nominally at 400–500 nm (light intensity 45 mW cm^{-2}) involving six lamps (Philips TL-D 18 W) After dilution of the reaction with THF, the mixture precipitated into some amount of methanol. The resulting polymer placed in vacuum oven for drying at room temperature.

Synthesis of pyrene-1-methanethiol (Py-SH)

Thiol functionalized pyrene (Py-SH) was synthesized according to the described procedure[1]. In a 50 mL round-bottom flask with a reflux condenser, pyrene methanol (1.000 g, 4.31 mmol) was added to a stirring solution of thiourea (0.328 g, 4.31 mmol) and 48% HBr (1400 μL) in 10 mL acetonitrile and 3 mL DCM. The solution was mixed approximately 30 min and refluxed at 90 °C for 9 h. Small quantities of the heated reaction mixture were placed into a cold (ice bath) 15% aqueous NaOH solution (50 mL) and stirred overnight at room temperature. While keeping the temperature below 10 °C, the sodium thiolate solution was cooled (ice bath) and acidified with 50 % aqueous H_2SO_4 to pH 2-3. Finally, the crude product was extracted with chloroform (3-15 mL), and extracts were washed with aqueous $NaHCO_3$ solution (10 mL), brine and then dried over Na_2SO_4. The obtained product was yellowish solid, yield= 55 % ^{1}H-NMR (400 MHz, DMSO-d6): δ 8.43–8.41 (m, 2H), 8.32–7.23 (m, 4H), 8.15 (s, 2H), 8.06 (m, 1H), 4.50 (s, 2H), 2.07 (s,1H).

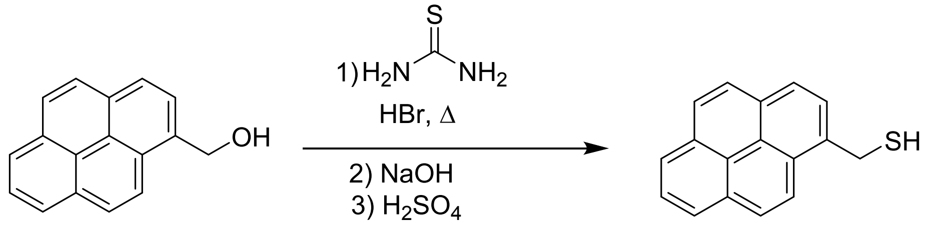

Scheme S1. Synthesis of pyrene-1-methanethiol (Py-SH)

Synthesis of pyrene functional PMMA-*b*-PIBVE

PMMA–I (1 eq) was dissolved in propylene carbonate (2 ml) and then, IBVE (100eq), $Ph_2I^+Br^-$ (0.5 eq) and $Mn_2(CO)_{10}$ (0.25 eq) were put into the Pyrex tube under dry nitrogen. The solution

was irradiated at room temperature for 90 mins and then quenched by pyrene-1-methanethiol (4 eq). Afterwards, the resulted solution precipitated into some amount of methanol. The obtained polymer placed in vacuum oven for drying at room temperature.

Characterization

^{1}H NMR were collected using 500 MHz on an Agilent VNMRS 500 spectrometer with $CDCl_3$ as solvent with $Si(CH_3)_4$ as an internal standard. Gel permeation chromatography (GPC) measurements were performed from a TOSOH EcoSEC GPC system equipped with an autosampler system, a temperature-controlled pump, a column oven, a refractive index (RI) detector, a purge and degasser unit, and a TSK gel superhZ2000 4.6 mm ID × 15 cm × 2 cm column. THF was the eluent at a flow rate of 1.0 mL min^{-1} at 40 °C. Both detectors calibration were done with polystyrene standards having narrow molecular-weight distribution. Differential scanning calorimetry (DSC) was performed on a Perkin-Elmer Diamond DSC from with a heating rate of 5°C min^{-1} under nitrogen flow. Fourier transform infrared (FT-IR) analyses were conducted on a Perkin-Elmer FTIR Spectrum One spectrometer. UV-vis spectra were recorded with a Shimadzu UV-1601 double-beam spectrometer equipped with a 50 W halogen lamp and a deuterium lamp which can operate between 190 and 1100 nm. All fluorescence spectra were recorded by using a PerkinElmer LS55 spectrometer performing between 200 and 900 nm wavelengths with a 10 nm slit width.

The gel permeation chromatograph (GPC) analysis using THF as an eluent was performed on a Shimadzu (Kyoto, Japan) LC-2030C Plus liquid chromatograph equipped with a Shodex (Tokyo, Japan) LF-804 column (300 × 8.0 mm; bead size = 6 μm; pore size = 3000 Å) and a Shodex KF-804L mixed gel column (300 × 8.0 mm; bead size = 7 μm; pore size = 1500 Å). The flow rate was 0.7 mL/min (40 °C). Sample detection was conducted using a Shimadzu refractive index detector (RID-20A). The column system was calibrated with standard PMMAs.

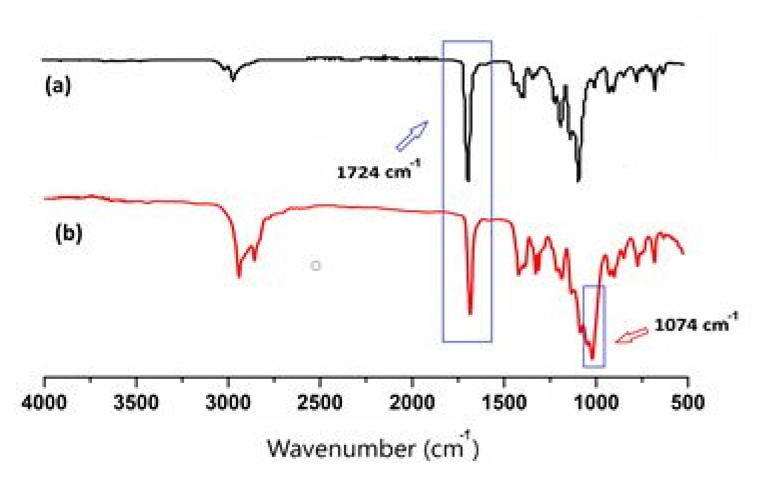

Figure S1. FTIR (ATR) spectra of (a) PMMA and (b) PMMA-*b*-PIBVE.

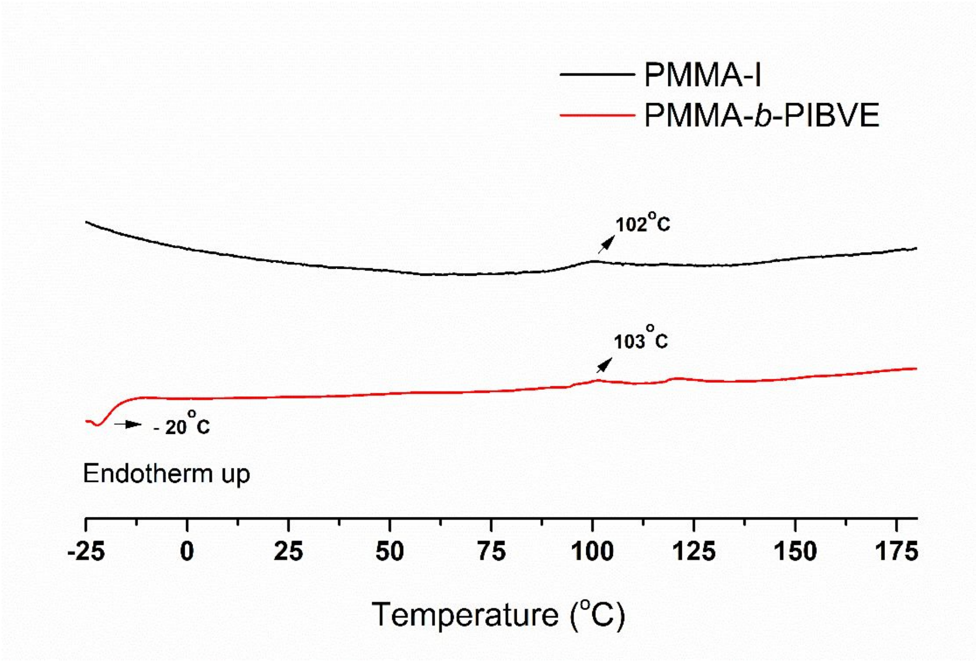

Figure S2. DSC traces of PMMA and PMMA-*b*-PIBVE.

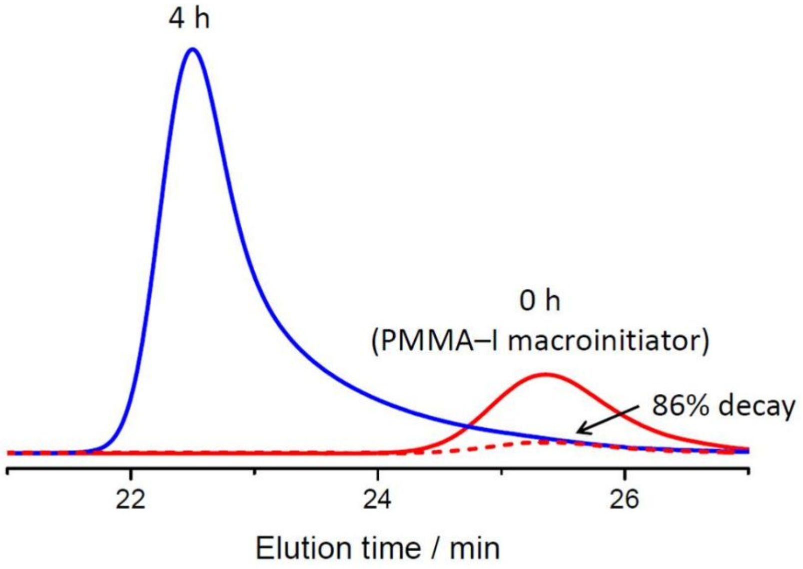

Figure S3. GPC chromatograms of PMMA–I (macroinitiator); MMA (200 eq), PMMA–I (1 eq), and BNI (2 eq) at 70 °C for t = 0 and 4 h.

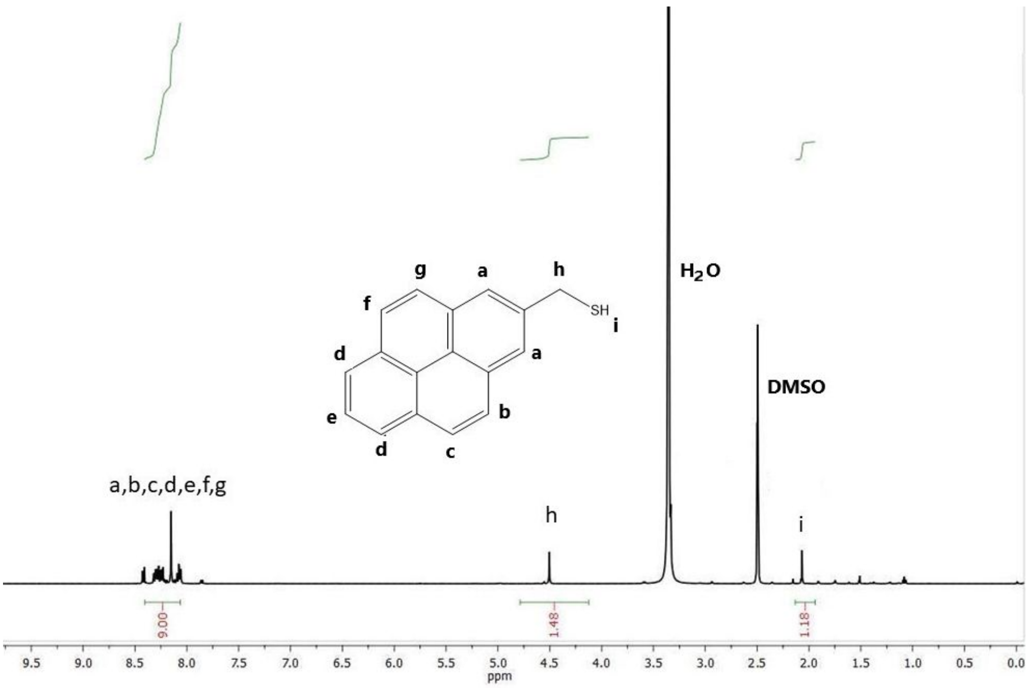

Figure S4. ^{1}H NMR spectrum of Py-SH.

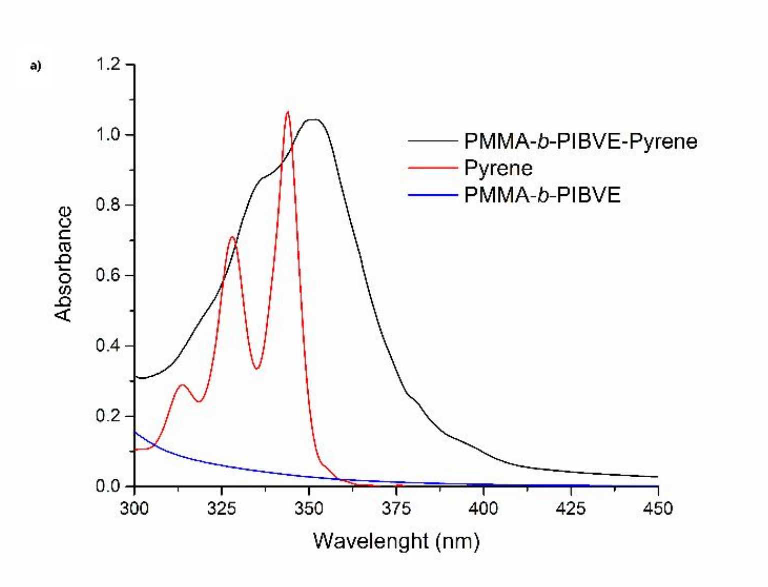

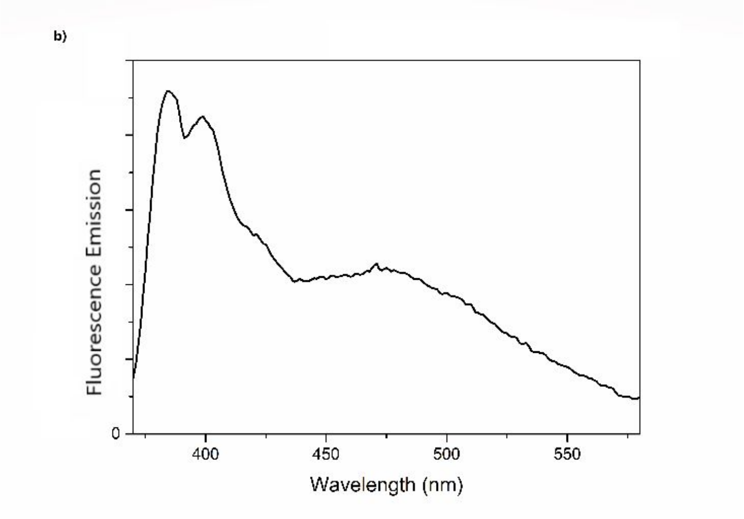

Figure S5. a) UV-vis spectra of PMMA-*b*-PIBVE (blue line); bare pyrene (red line) and PMMA-*b*-PIBVE-pyrene (black line). b) fluorescence emission spectrum of pyrene functional PMMA-*b*-PIBVE excited at 350 nm (5 x 10^{-5} mol L^{-1} in dichloromethane).

References

1. Tkachenko, B.A., Fokina, N.A., Chernish, L. V., Dahl, J.E.P., Liu, S., Carlson, R.M.K., Fokin, A.A., Schreiner, P.R., Functionalized nanodiamonds part 3: thiolation of tertiary/bridgehead alcohols , *Org. Lett.* **2006,** *8* (9), 1767–1770.

.
